# A real-world disproportionality analysis of mepolizumab based on the FDA adverse event reporting system

**DOI:** 10.3389/fphar.2023.1280490

**Published:** 2023-12-07

**Authors:** Huqun Li, Chongshu Wang, Aiping Deng, Cuilian Guo

**Affiliations:** ^1^ Department of Pharmacy, The Central Hospital of Wuhan, Tongji Medical College, Huazhong University of Science and Technology, Wuhan, China; ^2^ Department of Otolaryngology-Head and Neck Surgery, Tongji Hospital, Tongji Medical College, Huazhong University of Science and Technology, Wuhan, China

**Keywords:** mepolizumab, asthma, pharmacovigilance, disproportionality, pediatric, long term

## Abstract

**Background:** Mepolizumab has been approved by the FDA for add-on maintenance treatment of severe asthma with an eosinophilic phenotype. Real-world studies on mepolizumab-associated adverse events are limited. The present study aimed to explore mepolizumab-related adverse events based on the US Food and Drug Administration Adverse Event Reporting System (FAERS) database.

**Methods:** A disproportionality analysis was performed to assess the safety profile of mepolizumab based on the reports from the FAERS database between October 2015 and December 2022. Demographic information, the time to onset, the safety of long-term mepolizumab exposure as well as safety in pediatric patients were also investigated.

**Results:** A total of 736 significant preferred terms (PTs) were identified among the 13,497 mepolizumab-associated adverse events (AEs) reports collected from the FAERS database. The frequently reported AEs including dyspnea, fatigue, and headache were in line with drug instruction and previous studies. Unexpected significant AEs such as cough, malaise, and chest discomfort were also identified. Most AEs occurred within the first month after mepolizumab initiation. Pneumonia and wheezing were frequently reported in patients with long-term mepolizumab exposure as well as in the pediatric population.

**Conclusion:** Our results were consistent with the observations in previous clinical and real-world studies. New and unexpected AE signals of mepolizumab were also identified. Close attention should be paid to the long-term safety of mepolizumab as well as safety in the pediatric population. Prospective studies are required for optimal use of mepolizumab.

## Introduction

Asthma is a chronic and heterogeneous inflammatory disease that affects an estimated 358 million people all over the world (Khurana et al., 2019). Patients with severe asthma including severe eosinophilic asthma comprise 5%–10% of the total asthma population and have uncontrolled or partially controlled asthma despite historically inhaled corticosteroids and beta-agonists treatment (Pertzov et al., 2021). Recently, several biologics targeting various interleukin signaling pathways have been developed as an add-on treatment to standard-of-care asthma therapy with improved asthma control for patients with severe asthma (Charles et al., 2022; Hashimoto et al., 2022; Liu et al., 2023).

Interleukin 5 (IL-5) is a key regulator of eosinophil biology that has been implicated in the pathophysiology of severe eosinophilic asthma (Passalacqua, 2017). Mepolizumab, an anti-IL-5 monoclonal antibody, was approved by FDA in 2015 for add-on maintenance treatment of adult and pediatric patients aged 12 years and older with severe asthma and with an eosinophilic phenotype. Mepolizumab has also been approved for patients with eosinophilic diseases including chronic rhinosinusitis with nasal polyps (CRSwNP), eosinophilic granulomatosis with polyangiitis (EGPA), and hypereosinophilic syndrome (HES) (Roufosse et al., 2013; Wechsler et al., 2017). Although mepolizumab has demonstrated promising efficacy and good tolerance in clinical and real-world studies (Chupp et al., 2017; Khatri et al., 2019; Khurana et al., 2019; Pertzov et al., 2021), adverse events (AEs) such as headache and back pain were frequently reported in placebo-controlled trials. In addition, although mepolizumab has exhibited favorable long-term safety in clinical studies (Gleich et al., 2021; Maspero et al., 2022), long-term safety data beyond 1 year in real-world settings are lacking. Moreover, mepolizumab has been approved recently for severe eosinophilic asthma in patients aged 6 years and older in many countries (Drick et al., 2022). However, there is limited data available to evaluate the safety of mepolizumab in the pediatric population. As randomized clinical trials are sometimes very far from real-world conditions, comprehensive real-world pharmacovigilance to explore the adverse event profile of mepolizumab is urgently required.

The US Food and Drug Administration Adverse Event Report System (FAERS) database is a publicly accessible and the world’s largest pharmacovigilance database that receives drug-related AEs all over the world, including the United States and other countries (Shu et al., 2023). Therefore, it was particularly suitable for identifying potential associations between drugs and AEs due to the large size and global coverage. In addition, previous reports have shown notable accuracy of well-designed pharmacovigilance analysis based on the FAERS database (Harpaz et al., 2013; Raschi et al., 2021). Therefore, in the present study, we aim to conduct a comprehensive pharmacovigilance analysis of mepolizumab based on the FAERS database. Moreover, the long-term safety of mepolizumab, together with safety concerns in the pediatric population were also investigated for the first time. Our study indicated that mepolizumab should be prescribed with caution, and further studies are required to identify optimal treatment regimens, duration of treatment, and target patients. Our findings may serve as a valuable clinical reference.

## Materials and methods

### Data sources

**FIGURE 1:**
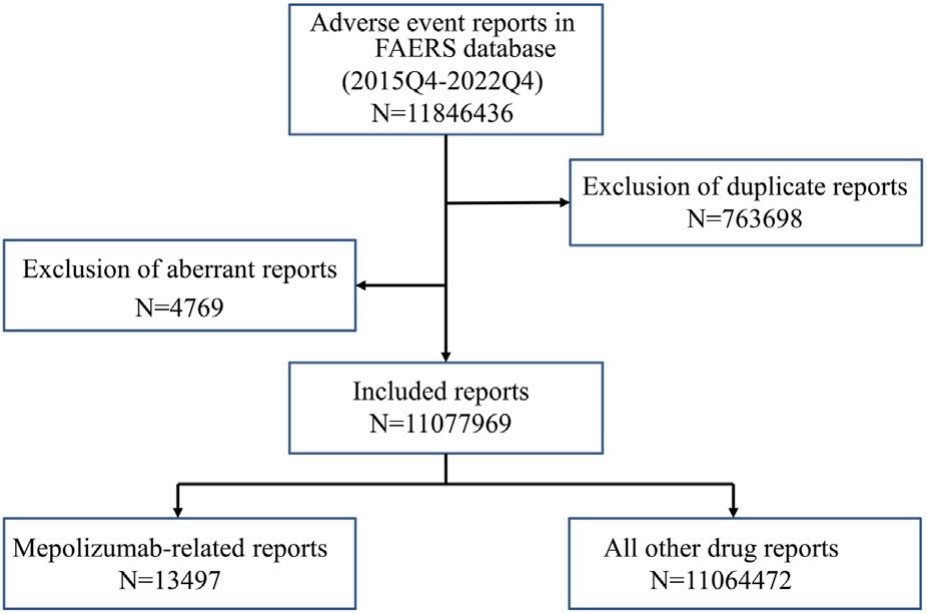
The flowchart of identifying mepolizumab related AEs in FAERS database. FAERS: Food and drug administration adverse event report system.

The present study was designed to evaluate whether a possible association exists between mepolizumab and an interest AE via a disproportionality analysis based on the FAERS database. Reports from the fourth quarter of 2015 (FDA marketing approval of mepolizumab) to the last quarter of 2022 (the most recent update of the FAERS database at the time this study was performed) were extracted to perform the disproportionality analysis. FAERS data were published quarterly by FDA and consisted of the following eight datasets: demographic and administrative information (DEMO), drug information (DRUG), adverse events (REAC), patient outcomes (OUTC), report sources (RPSR), start and end dates for reported drugs (THER), indications for use (INDI), and an additional deleted file (DELETED). As the FAERS inevitably contains duplicate reports, according to the FDA’s recommendations, we took the higher PRIMARYID when the CASEIDs were the same and the latest FDA_DT when the CASEIDs were the same to remove duplicates. In addition, the deleted cases were also removed. Both the generic name (mepolizumab) and brand names (nucala) were used to extract mepolizumab-associated reports in the DRUG file. In consideration of credibility, reports in which mepolizumab was deemed to cause adverse events with a role_cod of PS (Primary Suspect) were extracted for the disproportional analysis. AEs in the REAC file were recorded as the preferred term (PT) and subsequently system organ class (SOC) coded by the Medical Dictionary for Regulatory Activities (MedDRA) (version 26.0). The clinical characteristics of reports including gender, age, reporting countries, reporting year, reporter, outcomes, indications, therapy start time, and end time were collected and analyzed based on the available data. The time to onset of mepolizumab-associated AEs was calculated as the event onset date minus the therapy start date. Reports were excluded when an inaccurate date entry or an input error (EVENT_DT earlier than START_DT) occurred. The medication time of mepolizumab was calculated as the therapy end date minus the therapy start date. The flowchart of our study is shown in Figure 1.

### Statistical analysis

As the actual denominators were unclear, the incidence of AEs cannot be calculated by the FAERS database (Kinoshita et al., 2020). Therefore, as an effective and widely used method in pharmacovigilance studies, a disproportionality analysis was applied to identify potential signals of AEs related to mepolizumab in our study. The comparator group in disproportionality analysis is the background frequency of the same AE across all other drugs in FAERS compared with mepolizumab. The disproportionality analysis was performed using the reporting odds ratio (ROR) calculated by the case/non-case method (Lin et al., 2023). A higher ROR represented a stronger association between mepolizumab and AEs. ROR and 95% CI were calculated based on the 2 × 2 contingency tables with the formulas in Table 1.

**TABLE 1:** Calculation of reporting odds ratio (ROR) and 95% confidence interval (CI).

Drug category	Event of interest	All other events
Mepolizumab	a	b
All other drugs	c	d

ROR, ad/bc.

95% CI, e^ln(ROR)±1.96(1/a+1/b+1/c+1/d)^0.5^.

A significant signal was identified when the lower-bound 95% CI of the ROR was above 1.0 and the number of the cases was above 3. Data processing and analyses were performed by MYSQL 8.0, Microsoft Excel 2019, and GraphPad Prism 8. *p* < 0.05 was considered statistically significant.

## Result

### General characteristics

A total of 11,846,436 adverse events reports were submitted to the FAERS during the study period. After deduplication, 13,497 mepolizumab-associated reports were included in the study, and the general descriptions were presented in Table 2. The reports showed a generally increasing tendency year by year possibly due to the widespread use of mepolizumab. The mepolizumab-associated reports were more common in female than male patients (43.71% vs. 21.19%). Patients aged 18–60 years (13.92%) and elderly patients aged above 60 years (15.42%) accounted for most reports. A small proportion of reports were in children patients aged below 12 years (0.78%) and a smaller proportion in patients aged 12–18 years (0.48%). The most reported indication was asthma (82.48%), followed by EGPA (3.54%), HES (0.56%), Chronic obstructive pulmonary disease (COPD) (0.32%), and NP (0.28%). Regarding the report country, Canada (40.79%) submitted most reports, followed by America (29.70%), Japan (2.97%), Brazil (2.75%), and Australia (2.49%), respectively. The most frequently reported severe outcomes were OT (48.86%) and HO (24.58%).

**TABLE 2:** Clinical characteristics of mepolizumab associated reports from the FAERS database (October 2015 to December 2022).

Characteristics	Case Number, n	Proportion, %
Number of events	13497	
Gender		
Female	5900	43.71
Male	2860	21.19
Unknown	4737	35.10
Age (years)		
<12	105	0.78
12≤ and <18	65	0.48
18≤ and <60	1879	13.92
≥60	2081	15.42
Unknown	9367	69.40
Indications (top five)		
Asthma	11132	82.48
Eosinophilic granulomatosis with polyangiitis	478	3.54
Hypereosinophilic syndrome	75	0.56
Chronic obstructive pulmonary disease	43	0.32
Nasal polyps	38	0.28
Serious outcome		
Death (DE)	273	2.02
Life-threatening (LT)	69	0.51
Hospitalization-initial or prolonged (HO)	3318	24.58
Disability (DS)	58	0.43
Congenital anomaly (CA)	4	0.03
Other important medical events (OT)	6595	48.86
Required intervention (RI)	8	0.06
Reported countries (top five)		
Canada (CA)	5505	40.79
America (US)	4008	29.70
Japan (JP)	401	2.97
Brazil (BR)	371	2.75
Australia (AU)	336	2.49
Reporting year		
2022	4070	30.15
2021	2328	17.25
2020	2851	21.12
2019	2461	18.23
2018	601	4.45
2017	826	6.12
2016	356	2.64
2015Q4^a^	4	0.03

^a^ the fourth quarter of 2015.

### Signal detection

Table 3 lists the identified 83 significant PTs of interest. In the current study, AEs including dyspnoea (PT: 10013968), fatigue (PT: 10016256), headache (PT: 10019211), pyrexia (PT: 10037660), back pain (PT: 10003988), nasopharyngitis (PT: 10028810), arthralgia (PT: 10003239), asthenia (PT: 10003549), influenza (PT: 10022000), pruritus (PT: 10037087) were detected in data mining, which was consistent with the label for mepolizumab. Of note, unexpected AEs that were not listed in the FDA drug prescription were uncovered including pneumonia (PT: 10035664), wheezing (PT: 10047924), cough (PT: 10011224), malaise (PT: 10025482), chest discomfort (PT: 10008469), peripheral swelling (PT: 10048959), cataract (PT: 10007739), obstructive airways disorder (PT: 10061877) and so on. However, AEs such as abdominal pain, angioedema, diarrhea, dizziness, erythema, and flushing, which were listed in the drug label, did not meet the criteria for significant RORs in the present study.

**TABLE 3:** Signal strength of mepolizumab associated reports at the preferred terms level (*n* ≥ 100).

SOC	Preferred terms (PTs)	Cases (n)	ROR (95% two-sided CI)
Cardiac disorders	Cardiac disorder	124	2.11 (1.77–2.52)
	Myocardial infarction	107	1.44 (1.19–1.75)
Eye disorders	Cataract	181	4.04 (3.49–4.68)
General disorders and administration site conditions	Asthenia	348	1.29 (1.16–1.43)
	Chest discomfort	395	5.68 (5.14–6.28)
	Chest pain	255	2.22 (1.96–2.51)
	Chills	131	1.61 (1.35–1.91)
	Condition aggravated	551	2.24 (2.06–2.44)
	Fatigue	1026	1.69 (1.58–1.80)
	Ill-defined disorder	148	4.30 (3.65–5.06)
	Illness	146	2.44 (2.07–2.87)
	Influenza like illness	115	2.08 (1.73–2.50)
	Injection site pain	263	1.49 (1.32–1.68)
	Malaise	890	2.58 (2.41–2.76)
	Pain	598	1.15 (1.06–1.25)
	Peripheral swelling	190	1.23 (1.06–1.42)
	Pyrexia	520	2.09 (1.92–2.29)
	Secretion discharge	133	12.04 (10.14–14.31)
	Therapeutic product effect incomplete	670	9.83 (9.09–10.63)
Immune system disorders	Hypersensitivity	210	1.52 (1.33–1.74)
Infections and infestations	Bronchitis	242	3.88 (3.42–4.41)
	Cellulitis	111	2.84 (2.36–3.43)
	COVID-19	310	2.16 (1.93–2.42)
	Herpes zoster	172	3.55 (3.05–4.13)
	Infection	248	2.26 (2.00–2.57)
	Influenza	347	3.72 (3.35–4.14)
	Lower respiratory tract infection	184	4.40 (3.80–5.09)
	Nasopharyngitis	437	2.82 (2.56–3.10)
	Pneumonia	1807	7.92 (7.54–8.33)
	Respiratory tract infection	143	6.83 (5.78–8.05)
	Sinusitis	291	3.47 (3.09–3.90)
	Urinary tract infection	179	1.32 (1.14–1.53)
Injury, poisoning and procedural complications	Accidental exposure to product	228	3.57 (3.13–4.07)
	Contusion	104	1.39 (1.15–1.69)
	Exposure via skin contact	255	84.00 (73.77–95.65)
	Fall	385	1.55 (1.40–1.71)
	Inappropriate schedule of product administration	804	5.82 (5.42–6.25)
	Product dose omission issue	1265	4.85 (4.58–5.14)
	Underdose	173	2.75 (2.36–3.19)
	Wrong technique in device usage process	248	7.05 (6.22–8.00)
Investigations	Full blood count abnormal	169	8.04 (6.90–9.37)
	Blood pressure increased	271	2.09 (1.85–2.36)
	Heart rate increased	140	1.95 (1.65–2.30)
	Oxygen saturation decreased	182	4.13 (3.57–4.79)
Metabolism and nutrition disorders	Diabetes mellitus	126	2.51 (2.11–3.00)
Musculoskeletal and connective tissue disorders	Arthralgia	407	1.25 (1.13–1.38)
	Back pain	464	2.64 (2.41–2.90)
	Muscular weakness	103	1.30 (1.07–1.57)
	Myalgia	211	1.83 (1.59–2.09)
	Pain in extremity	339	1.46 (1.31–1.63)
Nervous system disorders	Cerebrovascular accident	118	1.29 (1.08–1.55)
	Headache	839	1.83 (1.71–1.97)
	Loss of consciousness	107	1.33 (1.10–1.61)
	Syncope	118	1.75 (1.46–2.09)
Product issues	Product availability issue	140	10.14 (8.58–11.99)
	Product complaint	274	14.08 (12.48–15.88)
Psychiatric disorders	Sleep disorder due to a general medical condition	591	59.67 (54.80–64.97)
Respiratory, thoracic and mediastinal disorders	Asthma	2804	41.37 (39.66–43.16)
	Asthmatic crisis	714	238.84 (219.45–259.93)
	Bronchospasm	101	11.33 (9.30–13.80)
	Chronic obstructive pulmonary disease	212	5.84 (5.10–6.69)
	Cough	1348	6.56 (6.20–6.94)
	Dysphonia	150	3.40 (2.89–3.99)
	Dyspnoea	2879	8.74 (8.38–9.10)
	Dyspnoea exertional	246	7.48 (6.59–8.49)
	Lung disorder	193	5.29 (4.59–6.10)
	Nasal congestion	247	5.30 (4.68–6.02)
	Obstructive airways disorder	178	14.57 (12.55–16.91)
	Oropharyngeal pain	182	2.38 (2.06–2.76)
	Productive cough	467	10.90 (9.93–11.96)
	Respiratory disorder	128	6.01 (5.05–7.16)
	Rhinorrhoea	163	2.94 (2.52–3.43)
	Sputum discoloured	214	20.18 (17.60–23.14)
	Wheezing	1445	33.99 (32.15–35.92)
Skin and subcutaneous tissue disorders	Pruritus	318	1.29 (1.15–1.44)
	Urticaria	167	1.52 (1.31–1.77)
Social circumstances	Loss of personal independence in daily activities	928	19.19 (17.94–20.52)
	Social problem	164	61.13 (52.12–71.70)
Surgical and medical procedures	Hospitalization	677	5.94 (5.49–6.41)
	Therapy interrupted	149	4.42 (3.76–5.19)
Vascular disorders	Hypertension	178	1.18 (1.02–1.37)

SOC, system organ class; ROR, reporting odds ratio; CI, confidence interval.

**FIGURE 2:**
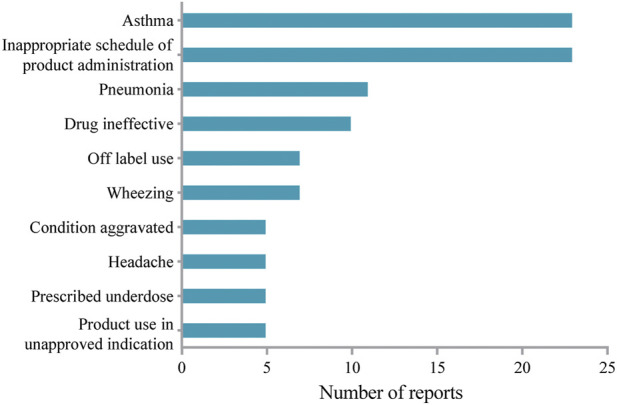
Top 10 reported adverse events associated with mepolizumab in pediatric population.

AEs reported in pediatric patients aged less than 12 years were extracted to study the safety profile in the pediatric population. The top 10 most frequently reported AEs were shown in Figure 2. Headache in the instructions was present among the top 10 most frequently reported AEs in the study. Of note, unexpected AEs such as wheezing and pneumonia were not included in the label. However, AEs including diarrhea, ear infection, and gastroenteritis as indicated in the label of mepolizumab were not recognized in the pediatric population.

**FIGURE 3:**
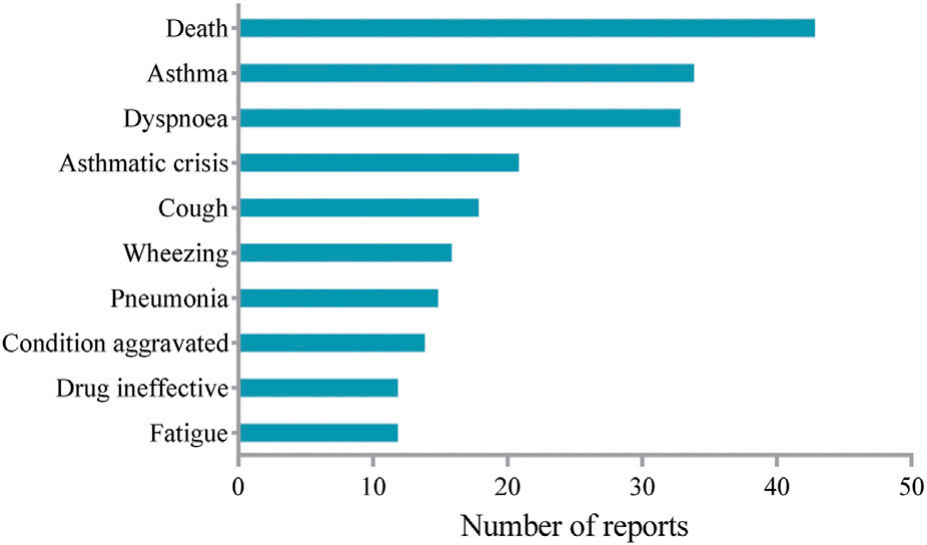
Top 10 reported adverse events during long term mepolizumab treatment.

AEs with a period above 52 weeks of mepolizumab medication were extracted to evaluate the long-term safety profile of mepolizumab. The top 10 most frequently reported AEs were summarized in Figure 3. Fatigue in the instructions was identified among the top 10 most frequently reported AEs. It is noteworthy that unexpected AEs including wheezing and pneumonia were uncovered in the label. However, AEs such as gastroenteritis and pharyngitis in the instruction were not observed after long-term mepolizumab treatment.

### Onset time of events

**FIGURE 4:**
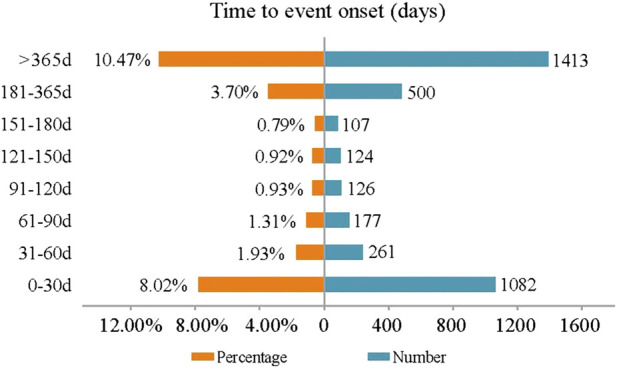
Time to onset of mepolizumab related AEs.

Figure 4 displays the onset times of mepolizumab-associated AEs. The median onset time was 685 days [interquartile range (IQR) 299–1176 days]. Most AEs occurred within the first 1 month (*n* = 1082, 8.02%) after mepolizumab initiation with almost half of the reports (*n* = 522, 3.88%) on the day of mepolizumab dosing. However, it should be noted that a considerable proportion of AEs (*n* = 1413, 10.47%) occurred after long-term (more than 1 year) mepolizumab treatment.

## Discussion

In the present study, we characterized the safety profile of mepolizumab based on the FAERS database. We identified new unexpected AEs associated with mepolizumab such as pneumonia and wheezing, and AEs listed in the drug label. Moreover, the long-term safety of mepolizumab as well as safety in pediatric patients was also evaluated in the current study. To our knowledge, the present study was the first pharmacovigilance analysis to explore the post-marketing safety of mepolizumab, providing a valuable reference for further optimal clinical use of mepolizumab.

In the present study, adult patients aged 18 years and older accounted for most AEs. Although mepolizumab has been approved for severe asthma in patients aged 6 years and older, and HES in patients aged 12 years and older, patients aged younger than 18 years accounted for a small proportion. Consistent with the instructions, mepolizumab has been prescribed mainly for asthma. However, a minor proportion of mepolizumab was used for COPD which was not included in the label. Despite the frequent coexistence and common pathophysiology of asthma and COPD (Maspero et al., 2022), previous studies have reported inconsistent results regarding the efficacy of mepolizumab for COPD (Drick et al., 2022; Ohnishi et al., 2022). Concerning the reporter countries, Canada and America accounted for most reports. Interestingly, China contributed to a minor proportion. Chronic rhinosinusitis with nasal polyposis (CRSwNP) is frequently associated with severe eosinophilic asthma and contributes to poor control of asthma (Detoraki et al., 2021). In white patients, 80%–90% of nasal polyps (NPs) are characterized by prominent eosinophilia with high amounts of IL-5. However, NPs of Chinese patients usually lack IL-5 and eotaxin expression which leads to lower numbers of tissue eosinophils (Gevaert et al., 2011). Furthermore, mepolizumab was approved recently in November 2021 in China for EGPA. Therefore, different races and ethnic backgrounds might be important underlying factors.

In line with the drug instructions, AEs such as nasopharyngitis, lower respiratory tract infection, and bronchitis were identified in the present study, which demonstrated the reliability of our research. However, new unexpected AEs including pneumonia, wheezing, lung disorder, and lung infection were also recognized as significant signals with high frequency. Moreover, in the sub-group analysis, pneumonia and wheezing were still frequently reported in patients after long-term mepolizumab treatment as well as in pediatric patients. Of note, pneumonia was also identified after long-term mepolizumab treatment while at a low rate in previous studies (Lugogo et al., 2016; Khatri et al., 2019).

Asthma affected approximately 11% of children aged 6–12 years (Nordlund et al., 2014). Moreover, mepolizumab was approved as an add-on treatment for severe eosinophilic asthma by the European Medicines Agency for patients 18 years and older in 2015, and later for patients 6 years and older in 2018 (Gupta et al., 2019). Therefore, we examined the mepolizumab-associated AEs in pediatric patients (aged less than 12 years old). Consistent with previous studies (Gupta et al., 2019), we identified mepolizumab-associated AEs such as bronchitis, headache, pyrexia, and so on, which were listed in the drug label. However, unexpected AEs including pneumonia and wheezing were also found in more than 7 reports in our study. Local injection-site reactions, which occurred with an incidence of 12% in adults in the COLUMBA study (Khatri et al., 2019), was not observed in our study as well as in Atul Gupta’s report (Gupta et al., 2019). Interestingly, local injection-site reaction was included in the drug label with an occurrence rate of 8% in severe asthma patients receiving mepolizumab. Moreover, we also identified mepolizumab-related anaphylaxis in children, which was not reported in adults (Lugogo et al., 2016; Khatri et al., 2019). Of note, inappropriate schedule of product administration, drug ineffective, and prescribed underdose were present among the frequently reported AEs, which suggested that inappropriate dose regimens might be common in pediatric patients due to the special physiology. Therefore, there exists an obvious difference in mepolizumab-associated AEs between adults and children. Mepolizumab should be prescribed for pediatric patients with more caution.

In the present study, asthma (exacerbation) and asthmatic crisis were identified among the most frequently reported AEs after long-term mepolizumab treatment, which were also most commonly reported in the COLUMBA study (Khatri et al., 2019). Consistently, no anaphylaxis was reported after long-term treatment of mepolizumab. However, pneumonia, which was frequently reported in the present study, was reported at a low rate in previous studies (Lugogo et al., 2016; Khatri et al., 2019). In addition, the most commonly reported AEs such as nasopharyngitis and upper respiratory tract infection in previous studies were present in limited reports in our study. It is noteworthy that death was present among the most frequently reported AEs during the long-term mepolizumab treatment. Given that these reports were voluntary, the relationship between mepolizumab and death requires further research and validation. Interestingly, herpes zoster infection, an opportunistic infection, was present in several reports in our study, which was also identified in previous studies (Khatri et al., 2019; Khurana et al., 2019). As mepolizumab significantly decreased eosinophils which might result in a loss of immune function regulation after long-term exposure, AEs occurred more likely compared with the shorter-term study. Therefore, further studies are needed to fully address the long-term safety profile of mepolizumab.

Concerning the onset time, most AEs occurred on the day of mepolizumab initiation, which was consistent with a previous multicenter and retrospective study (Montero-Pérez et al., 2019). Of note, AEs that occurred more than 365 days after mepolizumab initiation account for a considerable proportion. Therefore, a longer follow-up study is required to comprehensively address the mepolizumab-related AEs in clinical practice.

Severe eosinophilic asthma is characterized by chronic eosinophilic inflammation, mostly type 2 (T2) inflammation, in which IL-5 plays a central pathogenic role (Han et al., 2021). Moreover, Th2 response played a key role in most CRSwNP, especially in Western countries (Vinciguerra et al., 2022). Mepolizumab inhibited IL-5 signaling, reduced the Th2 response, and therefore alleviated the hypereosinophilia-related syndrome such as severe eosinophilic asthma. However, as a necessary part for viral immunity, the Th2 response inhibition raised the concern of an increased risk for viral infection among patients receiving mepolizumab. Indeed, we identified herpes zoster as a significant AE signal in our study, which is consistent with the warnings in the drug label of mepolizumab. Consistently, Sumita et al. identified opportunistic infections including herpes zoster infection after long-term mepolizumab treatment in patients with severe eosinophilic asthma (Khatri et al., 2019). Therefore, attention should be paid to the opportunistic infections associated with the mepolizumab treatment in clinical studies and real-world settings.

In the present study, we identified both allergic systemic reactions (rash and urticaria) and non-allergic systemic reactions (fatigue and paresthesia), which were consistent with the drug label. Of note, anaphylaxis identified in our study was reported to be mepolizumab unrelated in previous studies. It has been reported that humanized biologics such as mepolizumab have 90% of human component with a higher potential of immunogenicity compared with dupilumab that has 99% of human component (Bian et al., 2021). Therefore, the risk of anaphylaxis might be high for mepolizumab. Indeed, the post-marketing experience suggests a possible link between mepolizumab and hypersensitivity reactions including anaphylaxis in the drug label. Interestingly, mepolizumab-related anaphylaxis was identified in the pediatric population in our study as well as in previous studies, which suggested a predisposition to anaphylaxis for children. Therefore, further studies are needed to evaluate the systemic reactions during mepolizumab treatment, especially in pediatric patients.

We noted a recently published FAERS study of post-marketing safety of anti-IL-5 monoclonal antibodies including mepolizumab (Zou et al., 2023). Data mining methods mainly include frequency analysis and Bayesian analysis, and the sensitivity of Bayesian analysis is relatively lower than that of frequency analysis (Liu et al., 2022). In the recent study, both methods were used. Additionally, the AE of asthma and AE reports submitted by consumers were also excluded. In our study, only frequency analysis with higher sensitivity was applied to identify potential new and overlooked significant AEs. Therefore, AEs in the drug instructions such as arthralgia and AEs not included in the drug instructions such as peripheral swelling identified in our study were not observed in Shu-Peng Zou’s study. To some extent, our study provided a more comprehensive characterization of the AE signals for mepolizumab.

There are several limitations in our study. First, the voluntary reports in FAERS might be influenced by various factors such as FDA warnings which might lead to underreporting or overreporting of adverse events. Second, disproportionality analysis only provides a statistical evaluation of the signal strength but not real risk. A causal relationship between drug and AE does not require confirmation in the FAERS database, and some AE reports may have incomplete clinical and medical information such as age in the present study. Therefore, the causal relationship could not be established and requires further validation. Nevertheless, this real-world pharmacovigilance analysis provided wide monitoring and suspected AE signals of mepolizumab in an unselected population which might contribute to the rational use of mepolizumab in clinical practice. Moreover, prospective clinical studies and basic research are required to validate and confirm the AEs identified in the current study.

## Conclusion

The present study extends our knowledge of the safety concerns of mepolizumab in real-world clinical settings. Close attention should be paid to the AEs with strong real-world signals such as pneumonia and wheezing that are not listed in the label. Moreover, clinicians should take care of the long-term safety of mepolizumab for both children and adults, together with safety concerns in the pediatric population. In conclusion, it seems that mepolizumab should be prescribed with caution, and further clinical and real-world studies are required to identify optimal treatment regimens, duration of treatment, and target patients.

## Data Availability

The original contributions presented in the study are included in the article/Supplementary Material, further inquiries can be directed to the corresponding author.
