# Supplementary material for: A real-world disproportionality analysis of mepolizumab based on the FDA adverse event reporting system
Source: Front Pharmacol. 2023 Dec 7;14:1280490. doi: 10.3389/fphar.2023.1280490 (PMC10748586; doi:10.3389/fphar.2023.1280490)
Supplement: Supplementary file 1 [file Table1.DOCX]

**Supplementary Table 1** Signal strength of mepolizumab associated reports at the preferred terms level

| SOC | Preferred terms  (PT) | Cases  (n) | ROR | 95% CI | |
| --- | --- | --- | --- | --- | --- |
|  |  |  |  | Low bound | Up  bound |
| Blood and lymphatic system disorders | Hypercoagulation | 3 | 3.15 | 1.01 | 9.775 |
|  | Hypereosinophilic syndrome | 8 | 24.6 | 12.2 | 49.66 |
|  | Lymphadenopathy | 40 | 1.64 | 1.21 | 2.244 |
|  | Spontaneous haemorrhage | 4 | 4.75 | 1.78 | 12.7 |
| Cardiac disorders | Angina pectoris | 55 | 3.06 | 2.34 | 3.985 |
|  | Arrhythmia | 62 | 2.01 | 1.56 | 2.576 |
|  | Arteriosclerosis coronary artery | 7 | 2.27 | 1.08 | 4.771 |
|  | Atrial conduction time prolongation | 3 | 164 | 47.5 | 566.5 |
|  | Cardiac aneurysm | 3 | 4.37 | 1.4 | 13.59 |
|  | Cardiac disorder | 124 | 2.11 | 1.77 | 2.518 |
|  | Cardiac failure | 71 | 1.26 | 1 | 1.597 |
|  | Cardiac failure congestive | 75 | 2.25 | 1.79 | 2.82 |
|  | Cardiac valve disease | 8 | 2.19 | 1.1 | 4.392 |
|  | Cardiomyopathy | 18 | 2.17 | 1.37 | 3.455 |
|  | Cardiovascular symptom | 3 | 7.99 | 2.56 | 24.9 |
|  | Eosinophilic myocarditis | 6 | 12.9 | 5.78 | 29.01 |
|  | Heart valve incompetence | 7 | 3.1 | 1.47 | 6.505 |
|  | Left ventricular dysfunction | 10 | 2.1 | 1.13 | 3.901 |
|  | Mitral valve stenosis | 3 | 7.17 | 2.3 | 22.35 |
|  | Myocardial infarction | 107 | 1.44 | 1.19 | 1.746 |
|  | Postural orthostatic tachycardia syndrome | 3 | 3.53 | 1.14 | 10.99 |
|  | Right atrial enlargement | 3 | 11 | 3.51 | 34.31 |
|  | Tachycardia | 74 | 1.29 | 1.03 | 1.622 |
| Congenital, familial and genetic disorders | Asplenia | 3 | 43.9 | 13.7 | 140.4 |
|  | Cystic fibrosis | 25 | 4.62 | 3.12 | 6.848 |
|  | Factor V Leiden mutation | 8 | 28.2 | 13.9 | 57 |
|  | Factor VIII deficiency | 3 | 21.6 | 6.86 | 67.91 |
|  | Haemoglobinopathy | 3 | 154 | 44.8 | 527.7 |
|  | Syringomyelia | 3 | 15.9 | 5.06 | 49.75 |
|  | Thalassaemia | 5 | 34.2 | 14 | 83.61 |
| Ear and labyrinth disorders | Deafness | 33 | 1.72 | 1.22 | 2.425 |
|  | Ear congestion | 5 | 2.47 | 1.03 | 5.939 |
|  | Ear discomfort | 17 | 2.24 | 1.39 | 3.599 |
|  | Ear disorder | 13 | 3.4 | 1.97 | 5.867 |
|  | Ear pain | 41 | 2.64 | 1.94 | 3.588 |
|  | Ear pruritus | 8 | 3.26 | 1.63 | 6.528 |
|  | Otorrhoea | 8 | 5.22 | 2.6 | 10.46 |
|  | Vertigo positional | 5 | 3.74 | 1.55 | 9.007 |
| Endocrine disorders | Addison's disease | 6 | 6.78 | 3.03 | 15.14 |
|  | Adrenal insufficiency | 64 | 6.7 | 5.23 | 8.567 |
|  | Hyperthyroidism | 19 | 1.69 | 1.07 | 2.645 |
|  | Hypothalamic pituitary adrenal axis suppression | 3 | 12.6 | 4.01 | 39.26 |
| Eye disorders | Cataract | 181 | 4.04 | 3.49 | 4.68 |
|  | Conjunctival haemorrhage | 7 | 2.69 | 1.28 | 5.65 |
|  | Conjunctivitis allergic | 6 | 5.15 | 2.31 | 11.5 |
|  | Dacryostenosis acquired | 3 | 3.35 | 1.08 | 10.42 |
|  | Eye haemorrhage | 23 | 2.39 | 1.59 | 3.599 |
|  | Eye pruritus | 34 | 1.52 | 1.08 | 2.128 |
|  | Eye ulcer | 3 | 5.44 | 1.75 | 16.94 |
|  | Glaucoma | 38 | 2.57 | 1.87 | 3.531 |
|  | Ocular discomfort | 15 | 1.83 | 1.1 | 3.038 |
|  | Optic nerve compression | 3 | 21.6 | 6.86 | 67.91 |
|  | Retinal artery occlusion | 7 | 4.29 | 2.04 | 9.019 |
|  | Vitreous haemorrhage | 6 | 3.3 | 1.48 | 7.349 |
| Gastrointestinal disorders | Abdominal rigidity | 6 | 3 | 1.35 | 6.693 |
|  | Acute abdomen | 3 | 3.32 | 1.07 | 10.33 |
|  | Cheilitis | 7 | 2.37 | 1.13 | 4.974 |
|  | Dental discomfort | 6 | 10.2 | 4.56 | 22.84 |
|  | Intestinal mass | 5 | 4.75 | 1.97 | 11.43 |
|  | Lip discolouration | 6 | 6.67 | 2.99 | 14.89 |
|  | Lip pruritus | 3 | 4.39 | 1.41 | 13.66 |
|  | Mouth swelling | 11 | 2.12 | 1.17 | 3.828 |
|  | Odynophagia | 11 | 2.82 | 1.56 | 5.104 |
|  | Oesophageal compression | 3 | 60 | 18.6 | 193.8 |
|  | Oesophageal discomfort | 3 | 6.89 | 2.21 | 21.47 |
|  | Oesophageal obstruction | 3 | 4.81 | 1.55 | 14.98 |
|  | Oesophageal rupture | 5 | 9.09 | 3.77 | 21.95 |
|  | Oesophageal spasm | 4 | 3.43 | 1.28 | 9.161 |
|  | Oral mucosal discolouration | 3 | 6.24 | 2 | 19.44 |
|  | Pancreatitis relapsing | 5 | 9.98 | 4.13 | 24.1 |
|  | Paraesthesia oral | 22 | 2.39 | 1.58 | 3.64 |
|  | Salivary gland enlargement | 3 | 8.48 | 2.72 | 26.46 |
|  | Stomach mass | 3 | 4.1 | 1.32 | 12.75 |
|  | Swollen tongue | 41 | 2.22 | 1.63 | 3.014 |
|  | Teeth brittle | 7 | 6.7 | 3.18 | 14.1 |
|  | Tongue disorder | 13 | 2.83 | 1.64 | 4.88 |
|  | Tongue pruritus | 4 | 7.04 | 2.63 | 18.84 |
| General disorders and administration site conditions | Administration site bruise | 4 | 12 | 4.48 | 32.25 |
|  | Adverse drug reaction | 93 | 1.42 | 1.16 | 1.747 |
|  | Application site joint movement impairment | 3 | 2460 | 256 | 23650 |
|  | Asthenia | 348 | 1.29 | 1.16 | 1.433 |
|  | Chest discomfort | 395 | 5.68 | 5.14 | 6.278 |
|  | Chest pain | 255 | 2.22 | 1.96 | 2.51 |
|  | Chills | 131 | 1.61 | 1.35 | 1.91 |
|  | Condition aggravated | 551 | 2.24 | 2.06 | 2.438 |
|  | Crepitations | 12 | 4.66 | 2.64 | 8.219 |
|  | Crying | 33 | 1.47 | 1.04 | 2.067 |
|  | Discharge | 4 | 3.06 | 1.15 | 8.168 |
|  | Discomfort | 66 | 1.38 | 1.08 | 1.752 |
|  | Disease recurrence | 79 | 2.51 | 2.01 | 3.126 |
|  | Disease susceptibility | 4 | 12.6 | 4.68 | 33.74 |
|  | Exercise tolerance decreased | 19 | 4.24 | 2.7 | 6.66 |
|  | Facial discomfort | 8 | 25.4 | 12.6 | 51.41 |
|  | Fat tissue increased | 5 | 4.52 | 1.87 | 10.88 |
|  | Fatigue | 1026 | 1.69 | 1.58 | 1.797 |
|  | Feeling hot | 58 | 1.43 | 1.1 | 1.848 |
|  | Gait inability | 67 | 2.09 | 1.64 | 2.658 |
|  | Ill-defined disorder | 148 | 4.3 | 3.65 | 5.055 |
|  | Illness | 146 | 2.44 | 2.07 | 2.871 |
|  | Influenza like illness | 115 | 2.08 | 1.73 | 2.503 |
|  | Injection site discomfort | 22 | 2.89 | 1.9 | 4.395 |
|  | Injection site haematoma | 8 | 3.18 | 1.59 | 6.372 |
|  | Injection site pain | 263 | 1.49 | 1.32 | 1.68 |
|  | Injection site paraesthesia | 6 | 6.12 | 2.74 | 13.67 |
|  | Injection site urticaria | 28 | 2.07 | 1.43 | 2.997 |
|  | Malaise | 890 | 2.58 | 2.41 | 2.757 |
|  | Mucosal discolouration | 18 | 32.9 | 20.5 | 52.73 |
|  | Mucosal disorder | 3 | 3.66 | 1.18 | 11.38 |
|  | Multimorbidity | 4 | 5.82 | 2.17 | 15.55 |
|  | Nonspecific reaction | 13 | 4.33 | 2.51 | 7.464 |
|  | Obstruction | 8 | 2.57 | 1.28 | 5.151 |
|  | Oedema peripheral | 89 | 1.36 | 1.1 | 1.675 |
|  | Pain | 598 | 1.15 | 1.06 | 1.251 |
|  | Peripheral swelling | 190 | 1.23 | 1.06 | 1.417 |
|  | Polyp | 18 | 2.97 | 1.87 | 4.726 |
|  | Puncture site pain | 4 | 23.9 | 8.86 | 64.71 |
|  | Pyrexia | 520 | 2.09 | 1.92 | 2.287 |
|  | Secretion discharge | 133 | 12 | 10.1 | 14.31 |
|  | Sense of oppression | 7 | 7.07 | 3.36 | 14.88 |
|  | Swelling face | 81 | 1.93 | 1.55 | 2.403 |
|  | Symptom recurrence | 20 | 5.77 | 3.72 | 8.96 |
|  | Therapeutic product effect incomplete | 670 | 9.83 | 9.09 | 10.63 |
|  | Therapeutic product ineffective | 6 | 2.45 | 1.1 | 5.461 |
|  | Therapeutic response shortened | 36 | 1.62 | 1.17 | 2.249 |
|  | Therapeutic response unexpected | 91 | 2.37 | 1.93 | 2.909 |
|  | Thirst decreased | 4 | 9.4 | 3.51 | 25.19 |
|  | Vaccination failure | 7 | 5.43 | 2.58 | 11.41 |
| Hepatobiliary disorders | Bile duct stone | 7 | 3.02 | 1.44 | 6.354 |
|  | Cholangitis acute | 3 | 4.84 | 1.56 | 15.07 |
|  | Cholelithiasis | 32 | 1.63 | 1.15 | 2.307 |
|  | Hepatic mass | 5 | 3.05 | 1.27 | 7.331 |
|  | Hepatosplenomegaly | 4 | 2.68 | 1 | 7.146 |
|  | Jaundice cholestatic | 5 | 2.49 | 1.03 | 5.989 |
| Immune system disorders | Allergy to arthropod sting | 4 | 13.1 | 4.89 | 35.24 |
|  | Allergy to plants | 3 | 7.74 | 2.48 | 24.12 |
|  | Anaphylactic reaction | 85 | 2.46 | 1.98 | 3.042 |
|  | Anaphylactoid reaction | 8 | 4.24 | 2.12 | 8.498 |
|  | Eosinophilic granulomatosis with polyangiitis | 98 | 68.6 | 55.8 | 84.39 |
|  | Food allergy | 16 | 3.12 | 1.91 | 5.097 |
|  | Hypersensitivity | 210 | 1.52 | 1.33 | 1.742 |
|  | Immune system disorder | 31 | 2.86 | 2.01 | 4.074 |
|  | Immunosuppression | 24 | 3.77 | 2.52 | 5.626 |
|  | Mite allergy | 6 | 9.55 | 4.27 | 21.37 |
|  | Multiple allergies | 62 | 10.5 | 8.18 | 13.51 |
|  | Perfume sensitivity | 4 | 5.21 | 1.95 | 13.94 |
|  | Sarcoidosis | 12 | 3.46 | 1.96 | 6.093 |
|  | Seasonal allergy | 26 | 2.05 | 1.39 | 3.013 |
|  | Serum sickness | 5 | 3.52 | 1.46 | 8.473 |
| Infections and infestations | Acute sinusitis | 4 | 3.16 | 1.18 | 8.436 |
|  | Appendicitis | 12 | 1.8 | 1.02 | 3.172 |
|  | Arthritis infective | 27 | 6.94 | 4.75 | 10.15 |
|  | Aspergillus infection | 26 | 4.31 | 2.93 | 6.333 |
|  | Atypical pneumonia | 8 | 3.24 | 1.62 | 6.486 |
|  | Bacterial abdominal infection | 4 | 38.6 | 14.2 | 105.2 |
|  | Bacterial infection | 38 | 2.95 | 2.14 | 4.052 |
|  | Bronchitis | 242 | 3.88 | 3.42 | 4.412 |
|  | Bronchopulmonary aspergillosis | 16 | 2.76 | 1.69 | 4.508 |
|  | Bronchopulmonary aspergillosis allergic | 15 | 16.8 | 10.1 | 27.97 |
|  | Cellulitis | 111 | 2.84 | 2.36 | 3.428 |
|  | Cellulitis orbital | 7 | 19.3 | 9.13 | 40.91 |
|  | Chikungunya virus infection | 5 | 9.17 | 3.8 | 22.15 |
|  | Chronic sinusitis | 33 | 10.2 | 7.23 | 14.38 |
|  | Coronavirus infection | 75 | 11.5 | 9.15 | 14.46 |
|  | COVID-19 | 310 | 2.16 | 1.93 | 2.418 |
|  | COVID-19 pneumonia | 25 | 2.25 | 1.52 | 3.331 |
|  | Cystitis | 49 | 1.81 | 1.37 | 2.396 |
|  | Dengue fever | 10 | 6.53 | 3.5 | 12.17 |
|  | Diverticulitis | 67 | 2.78 | 2.19 | 3.538 |
|  | Ear infection | 42 | 1.9 | 1.4 | 2.573 |
|  | Erysipelas | 9 | 2.13 | 1.11 | 4.101 |
|  | Fungal oesophagitis | 5 | 10 | 4.14 | 24.16 |
|  | Fungal pharyngitis | 6 | 20.9 | 9.31 | 47.09 |
|  | Gastric infection | 13 | 4.06 | 2.35 | 6.995 |
|  | Gingival abscess | 3 | 5.24 | 1.69 | 16.32 |
|  | Gingivitis | 8 | 2.05 | 1.02 | 4.106 |
|  | Haemophilus infection | 5 | 7.54 | 3.12 | 18.18 |
|  | Helminthic infection | 3 | 14.7 | 4.7 | 46.14 |
|  | Herpes ophthalmic | 4 | 3.85 | 1.44 | 10.29 |
|  | Herpes zoster | 172 | 3.55 | 3.05 | 4.128 |
|  | Infected cyst | 6 | 6.06 | 2.71 | 13.53 |
|  | Infected skin ulcer | 6 | 4.07 | 1.82 | 9.078 |
|  | Infection | 248 | 2.26 | 2 | 2.569 |
|  | Influenza | 347 | 3.72 | 3.35 | 4.144 |
|  | Kidney infection | 48 | 2.86 | 2.15 | 3.798 |
|  | Labyrinthitis | 5 | 2.58 | 1.07 | 6.212 |
|  | Laryngitis | 15 | 1.87 | 1.13 | 3.107 |
|  | Latent tuberculosis | 13 | 7.31 | 4.23 | 12.62 |
|  | Localised infection | 42 | 2.04 | 1.51 | 2.768 |
|  | Lower respiratory tract infection | 184 | 4.4 | 3.8 | 5.089 |
|  | Lower respiratory tract infection bacterial | 8 | 16 | 7.93 | 32.15 |
|  | Lower respiratory tract infection viral | 5 | 10.4 | 4.3 | 25.08 |
|  | Lung abscess | 11 | 7.37 | 4.07 | 13.35 |
|  | Lung infection | 177 | 12.2 | 10.5 | 14.16 |
|  | Lyme disease | 7 | 2.55 | 1.21 | 5.359 |
|  | Mastitis | 5 | 2.87 | 1.19 | 6.905 |
|  | Mycobacterial infection | 6 | 4.18 | 1.87 | 9.326 |
|  | Mycotic endophthalmitis | 4 | 36.9 | 13.5 | 100.4 |
|  | Nasopharyngitis | 437 | 2.82 | 2.56 | 3.102 |
|  | Oesophageal candidiasis | 11 | 3.98 | 2.2 | 7.205 |
|  | Ophthalmic herpes simplex | 4 | 8.52 | 3.18 | 22.82 |
|  | Ophthalmic herpes zoster | 8 | 4.78 | 2.39 | 9.584 |
|  | Oral infection | 7 | 2.66 | 1.27 | 5.593 |
|  | Parainfluenzae virus infection | 4 | 2.93 | 1.1 | 7.817 |
|  | Parotitis | 3 | 4.48 | 1.44 | 13.94 |
|  | Pharyngitis | 24 | 2.14 | 1.43 | 3.198 |
|  | Pneumonia | 1630 | 7.47 | 7.1 | 7.873 |
|  | Pneumonia bacterial | 29 | 4.32 | 3 | 6.221 |
|  | Pneumonia fungal | 7 | 2.68 | 1.28 | 5.627 |
|  | Pneumonia pseudomonal | 10 | 7.92 | 4.25 | 14.76 |
|  | Pneumonia respiratory syncytial viral | 3 | 5.79 | 1.86 | 18.02 |
|  | Pneumonia viral | 10 | 3.75 | 2.01 | 6.972 |
|  | Poliomyelitis | 3 | 50.2 | 15.6 | 161.1 |
|  | Pseudomonas infection | 24 | 4.26 | 2.85 | 6.363 |
|  | Psoas abscess | 3 | 7.28 | 2.34 | 22.68 |
|  | Pulmonary mycosis | 8 | 10.9 | 5.41 | 21.83 |
|  | Pulpitis dental | 4 | 4.49 | 1.68 | 12.01 |
|  | Pyelonephritis | 16 | 2.39 | 1.46 | 3.897 |
|  | Pyelonephritis acute | 5 | 3.33 | 1.38 | 8.017 |
|  | Respiratory tract infection | 143 | 6.83 | 5.78 | 8.053 |
|  | Respiratory tract infection fungal | 5 | 11.3 | 4.66 | 27.23 |
|  | Rhinitis | 76 | 11.1 | 8.82 | 13.89 |
|  | Sialoadenitis | 10 | 9.44 | 5.06 | 17.61 |
|  | Sinusitis | 291 | 3.47 | 3.09 | 3.898 |
|  | Sinusitis bacterial | 8 | 9.71 | 4.83 | 19.49 |
|  | Sinusitis fungal | 4 | 5.69 | 2.13 | 15.23 |
|  | Skin infection | 16 | 1.76 | 1.08 | 2.883 |
|  | Sputum purulent | 30 | 53.6 | 37 | 77.55 |
|  | Staphylococcal infection | 33 | 1.53 | 1.09 | 2.16 |
|  | Suspected COVID-19 | 37 | 8.57 | 6.2 | 11.86 |
|  | Upper respiratory tract infection | 85 | 2.3 | 1.86 | 2.848 |
|  | Urinary tract infection | 179 | 1.32 | 1.14 | 1.533 |
|  | Varicella | 6 | 3.58 | 1.61 | 7.992 |
|  | Viral disease carrier | 3 | 154 | 44.8 | 527.7 |
|  | Viral infection | 88 | 3.6 | 2.92 | 4.438 |
|  | Viral upper respiratory tract infection | 20 | 3.27 | 2.11 | 5.076 |
|  | Wound infection | 35 | 4.66 | 3.34 | 6.504 |
|  | Zika virus infection | 3 | 27 | 8.56 | 85.39 |
| Injury, poisoning and procedural complications | Accident | 16 | 1.75 | 1.07 | 2.853 |
|  | Accident at home | 3 | 7.3 | 2.34 | 22.75 |
|  | Accidental exposure to product | 228 | 3.57 | 3.13 | 4.065 |
|  | Accidental underdose | 17 | 2.05 | 1.27 | 3.295 |
|  | Anaesthetic complication neurological | 4 | 11.3 | 4.22 | 30.34 |
|  | Animal scratch | 5 | 6.11 | 2.53 | 14.73 |
|  | Bone fragmentation | 6 | 21.8 | 9.68 | 48.99 |
|  | Breast injury | 5 | 29.9 | 12.3 | 73.07 |
|  | Chest injury | 18 | 8.77 | 5.51 | 13.96 |
|  | Circumstance or information capable of leading to medication error | 45 | 2.17 | 1.62 | 2.905 |
|  | Contusion | 104 | 1.39 | 1.15 | 1.685 |
|  | Drug exposure before pregnancy | 3 | 7.76 | 2.49 | 24.19 |
|  | Exposure to allergen | 3 | 14.3 | 4.57 | 44.79 |
|  | Exposure to SARS-CoV-2 | 11 | 5.24 | 2.9 | 9.488 |
|  | Exposure via skin contact | 255 | 84 | 73.8 | 95.65 |
|  | Facial bones fracture | 10 | 3.05 | 1.64 | 5.679 |
|  | Fall | 385 | 1.55 | 1.4 | 1.712 |
|  | Forearm fracture | 3 | 5.83 | 1.87 | 18.15 |
|  | Foreign body in gastrointestinal tract | 3 | 8.01 | 2.57 | 24.98 |
|  | Foreign body in skin or subcutaneous tissue | 3 | 68.3 | 21 | 221.9 |
|  | Frostbite | 3 | 14 | 4.46 | 43.76 |
|  | Hip fracture | 37 | 1.61 | 1.17 | 2.228 |
|  | Immunisation reaction | 3 | 5.88 | 1.89 | 18.32 |
|  | Inappropriate schedule of product administration | 804 | 5.82 | 5.42 | 6.251 |
|  | Incision site discharge | 3 | 17.8 | 5.68 | 55.96 |
|  | Incorrect product administration duration | 50 | 2.72 | 2.06 | 3.594 |
|  | Injection related reaction | 13 | 11.3 | 6.54 | 19.56 |
|  | Joint dislocation | 14 | 1.75 | 1.03 | 2.952 |
|  | Joint injury | 29 | 1.68 | 1.17 | 2.424 |
|  | Ligament injury | 3 | 3.72 | 1.2 | 11.55 |
|  | Ligament sprain | 17 | 1.84 | 1.14 | 2.957 |
|  | Limb injury | 86 | 3.57 | 2.89 | 4.421 |
|  | Lower limb fracture | 33 | 2.43 | 1.73 | 3.422 |
|  | Lumbar vertebral fracture | 13 | 3.87 | 2.25 | 6.682 |
|  | Muscle rupture | 13 | 4.53 | 2.62 | 7.813 |
|  | Patella fracture | 6 | 3.55 | 1.59 | 7.917 |
|  | Procedural complication | 12 | 3.49 | 1.98 | 6.149 |
|  | Product administration interrupted | 10 | 2.55 | 1.37 | 4.739 |
|  | Product dispensing issue | 4 | 2.86 | 1.07 | 7.646 |
|  | Product dose omission | 379 | 3.76 | 3.39 | 4.163 |
|  | Product dose omission issue | 886 | 5.24 | 4.89 | 5.609 |
|  | Product preparation issue | 12 | 2.93 | 1.66 | 5.16 |
|  | Radius fracture | 7 | 4.44 | 2.11 | 9.341 |
|  | Rib fracture | 62 | 3.89 | 3.03 | 4.994 |
|  | Road traffic accident | 49 | 1.79 | 1.35 | 2.37 |
|  | Sciatic nerve injury | 3 | 4.2 | 1.35 | 13.05 |
|  | Scratch | 16 | 2.22 | 1.36 | 3.628 |
|  | Skin injury | 8 | 2.96 | 1.48 | 5.937 |
|  | Skin laceration | 22 | 2.57 | 1.69 | 3.911 |
|  | Skin wound | 9 | 7.27 | 3.77 | 14.01 |
|  | Spinal column injury | 5 | 2.49 | 1.03 | 5.982 |
|  | Spinal cord injury | 4 | 2.85 | 1.07 | 7.613 |
|  | Spinal fracture | 31 | 2.04 | 1.43 | 2.9 |
|  | Surgical procedure repeated | 3 | 25.1 | 7.96 | 79.19 |
|  | Thoracic vertebral fracture | 6 | 2.74 | 1.23 | 6.104 |
|  | Traumatic fracture | 3 | 5.77 | 1.85 | 17.98 |
|  | Traumatic lung injury | 12 | 7.75 | 4.39 | 13.68 |
|  | Traumatic shock | 4 | 50.5 | 18.4 | 138.5 |
|  | Underdose | 173 | 2.75 | 2.36 | 3.191 |
|  | Upper limb fracture | 25 | 1.51 | 1.02 | 2.231 |
|  | Uterine injury | 6 | 70.3 | 30.5 | 161.8 |
|  | Vaccination complication | 7 | 6.38 | 3.03 | 13.42 |
|  | Wrist fracture | 18 | 1.98 | 1.24 | 3.138 |
|  | Wrong schedule | 3 | 3.18 | 1.02 | 9.876 |
|  | Wrong technique in device usage process | 248 | 7.05 | 6.22 | 8.001 |
| Investigations | Alanine aminotransferase abnormal | 5 | 2.66 | 1.11 | 6.41 |
|  | Antineutrophil cytoplasmic antibody increased | 3 | 24.4 | 7.72 | 76.8 |
|  | Antineutrophil cytoplasmic antibody positive | 4 | 9.79 | 3.65 | 26.24 |
|  | Auscultation | 3 | 145 | 42.4 | 493.8 |
|  | Biopsy bone marrow | 7 | 22.5 | 10.6 | 47.72 |
|  | Biopsy endometrium abnormal | 4 | 1640 | 300 | 8955 |
|  | Biopsy lung | 4 | 12.6 | 4.7 | 33.87 |
|  | Biopsy tongue | 3 | 129 | 38.3 | 437.6 |
|  | Blood alkaline phosphatase abnormal | 3 | 5.09 | 1.64 | 15.85 |
|  | Blood cholesterol abnormal | 9 | 2.77 | 1.44 | 5.33 |
|  | Blood count abnormal | 169 | 8.04 | 6.9 | 9.365 |
|  | Blood electrolytes abnormal | 3 | 3.92 | 1.26 | 12.18 |
|  | Blood gases | 3 | 273 | 74 | 1010 |
|  | Blood immunoglobulin E decreased | 7 | 48.2 | 22.5 | 103.4 |
|  | Blood immunoglobulin E increased | 31 | 13.5 | 9.49 | 19.31 |
|  | Blood pressure abnormal | 25 | 1.58 | 1.07 | 2.336 |
|  | Blood pressure increased | 271 | 2.09 | 1.85 | 2.357 |
|  | Blood test abnormal | 31 | 2.59 | 1.82 | 3.69 |
|  | Blood urine | 5 | 10.2 | 4.22 | 24.64 |
|  | Body temperature abnormal | 9 | 4.91 | 2.55 | 9.46 |
|  | Breath sounds | 4 | 26.5 | 9.77 | 71.6 |
|  | Breath sounds abnormal | 48 | 11.2 | 8.45 | 14.94 |
|  | Breath sounds absent | 3 | 39 | 12.3 | 124.3 |
|  | Carbon dioxide increased | 4 | 4.09 | 1.53 | 10.92 |
|  | Cardiac murmur | 14 | 2.83 | 1.68 | 4.788 |
|  | Cardiac output decreased | 3 | 5.49 | 1.76 | 17.09 |
|  | Chest X-ray abnormal | 14 | 7.32 | 4.32 | 12.39 |
|  | Colonoscopy | 6 | 3.65 | 1.64 | 8.147 |
|  | Computerised tomogram abnormal | 6 | 5.06 | 2.27 | 11.3 |
|  | Coronavirus test positive | 55 | 45.5 | 34.7 | 59.72 |
|  | Emergency care examination | 4 | 10.1 | 3.78 | 27.14 |
|  | Enzyme level increased | 5 | 6.54 | 2.71 | 15.77 |
|  | Eosinophil count abnormal | 41 | 58 | 42.3 | 79.68 |
|  | Eosinophil count decreased | 31 | 22.7 | 15.9 | 32.48 |
|  | Eosinophil count increased | 64 | 9.61 | 7.51 | 12.3 |
|  | FEV1/FVC ratio abnormal | 3 | 76.9 | 23.5 | 251.1 |
|  | FEV1/FVC ratio decreased | 3 | 23 | 7.3 | 72.42 |
|  | Forced expiratory volume abnormal | 7 | 46.3 | 21.6 | 99.16 |
|  | Full blood count abnormal | 52 | 6.43 | 4.89 | 8.451 |
|  | Heart rate increased | 140 | 1.95 | 1.65 | 2.298 |
|  | Heart rate irregular | 30 | 1.57 | 1.1 | 2.254 |
|  | Heart sounds | 6 | 45.1 | 19.8 | 102.7 |
|  | Heart sounds abnormal | 3 | 6.61 | 2.12 | 20.6 |
|  | Immunoglobulins increased | 3 | 9.92 | 3.18 | 30.97 |
|  | Interleukin level increased | 3 | 10.3 | 3.31 | 32.28 |
|  | Monocyte count increased | 9 | 2.75 | 1.43 | 5.286 |
|  | Muscle strength abnormal | 3 | 10.5 | 3.35 | 32.69 |
|  | Mycobacterium test positive | 4 | 27.3 | 10.1 | 74.03 |
|  | Nerve conduction studies | 3 | 820 | 165 | 4063 |
|  | Oxygen saturation | 5 | 19.7 | 8.12 | 47.87 |
|  | Oxygen saturation abnormal | 38 | 13.8 | 10 | 19.09 |
|  | Oxygen saturation decreased | 182 | 4.13 | 3.57 | 4.788 |
|  | Peak expiratory flow rate abnormal | 4 | 63.1 | 22.8 | 174.4 |
|  | Peak expiratory flow rate decreased | 14 | 36 | 21.1 | 61.52 |
|  | Plethysmography | 3 | 87.9 | 26.7 | 289 |
|  | Polymerase chain reaction positive | 3 | 7.59 | 2.44 | 23.67 |
|  | Pulmonary function test abnormal | 16 | 9.29 | 5.68 | 15.21 |
|  | Pulmonary function test decreased | 29 | 6.37 | 4.42 | 9.18 |
|  | Respiratory rate decreased | 13 | 6.07 | 3.52 | 10.48 |
|  | Respiratory rate increased | 29 | 4.15 | 2.88 | 5.973 |
|  | SARS-CoV-2 test negative | 12 | 16.7 | 9.45 | 29.66 |
|  | SARS-CoV-2 test positive | 57 | 4.13 | 3.18 | 5.355 |
|  | Spirometry abnormal | 9 | 15.1 | 7.79 | 29.14 |
|  | Sputum abnormal | 5 | 9.67 | 4 | 23.36 |
|  | Sputum culture | 3 | 57.2 | 17.7 | 184.4 |
|  | Sputum culture positive | 4 | 13 | 4.85 | 34.95 |
|  | Total lung capacity abnormal | 5 | 22.9 | 9.42 | 55.72 |
|  | Total lung capacity decreased | 18 | 13.1 | 8.19 | 20.8 |
|  | Vital capacity decreased | 11 | 21.5 | 11.8 | 39.2 |
|  | Weight abnormal | 7 | 3.24 | 1.54 | 6.799 |
| Metabolism and nutrition disorders | Diabetes mellitus | 126 | 2.51 | 2.11 | 2.997 |
|  | Glucose tolerance impaired | 10 | 2.61 | 1.4 | 4.86 |
|  | Gout | 25 | 1.87 | 1.26 | 2.763 |
|  | Marasmus | 6 | 6.43 | 2.88 | 14.36 |
|  | Obesity | 27 | 2.27 | 1.55 | 3.311 |
|  | Starvation | 5 | 10.6 | 4.38 | 25.6 |
|  | Steroid diabetes | 6 | 4.82 | 2.16 | 10.76 |
|  | Type 2 diabetes mellitus | 49 | 2.47 | 1.87 | 3.273 |
| Musculoskeletal and connective tissue disorders | Arthralgia | 407 | 1.25 | 1.13 | 1.383 |
|  | Arthritis | 85 | 1.33 | 1.07 | 1.644 |
|  | Back pain | 464 | 2.64 | 2.41 | 2.901 |
|  | Bursitis | 14 | 1.76 | 1.04 | 2.969 |
|  | Crystal arthropathy | 4 | 25.6 | 9.47 | 69.33 |
|  | Fracture pain | 5 | 12.2 | 5.03 | 29.43 |
|  | Intervertebral disc compression | 3 | 3.68 | 1.18 | 11.43 |
|  | Intervertebral disc degeneration | 13 | 1.93 | 1.12 | 3.321 |
|  | Kyphosis | 7 | 6.09 | 2.9 | 12.82 |
|  | Limb discomfort | 45 | 1.51 | 1.12 | 2.02 |
|  | Mobility decreased | 92 | 1.55 | 1.27 | 1.908 |
|  | Muscle disorder | 12 | 2.26 | 1.28 | 3.984 |
|  | Muscle mass | 6 | 16 | 7.14 | 35.96 |
|  | Muscular weakness | 103 | 1.3 | 1.07 | 1.574 |
|  | Musculoskeletal chest pain | 23 | 1.88 | 1.25 | 2.836 |
|  | Myalgia | 211 | 1.83 | 1.59 | 2.092 |
|  | Neck pain | 59 | 1.38 | 1.07 | 1.781 |
|  | Oligoarthritis | 3 | 15 | 4.79 | 46.99 |
|  | Osteoarthritis | 50 | 1.49 | 1.13 | 1.962 |
|  | Osteoporosis | 61 | 1.47 | 1.14 | 1.885 |
|  | Pain in extremity | 339 | 1.46 | 1.31 | 1.63 |
|  | Polymyalgia rheumatica | 22 | 11.3 | 7.4 | 17.17 |
|  | Sjogren's syndrome | 10 | 2.41 | 1.29 | 4.479 |
|  | Soft tissue swelling | 3 | 4.23 | 1.36 | 13.14 |
|  | Spinal flattening | 5 | 39.1 | 15.9 | 95.79 |
|  | Spinal pain | 31 | 2.66 | 1.87 | 3.78 |
|  | Synovial cyst | 10 | 2.3 | 1.24 | 4.279 |
| Neoplasms benign, malignant and unspecified (incl cysts and polyps) | Acoustic neuroma | 4 | 13.6 | 5.05 | 36.41 |
|  | Adrenal neoplasm | 3 | 9.68 | 3.1 | 30.23 |
|  | Bladder neoplasm | 4 | 3.05 | 1.14 | 8.13 |
|  | Brain neoplasm | 13 | 1.9 | 1.1 | 3.277 |
|  | Breast cancer male | 5 | 12.2 | 5.03 | 29.43 |
|  | Colon cancer | 24 | 2.11 | 1.41 | 3.152 |
|  | Endometrial cancer | 5 | 3.79 | 1.57 | 9.124 |
|  | Glioblastoma | 5 | 4.96 | 2.06 | 11.96 |
|  | Leiomyoma | 11 | 20.9 | 11.5 | 38.01 |
|  | Lung neoplasm | 7 | 3.76 | 1.79 | 7.906 |
|  | Neoplasm malignant | 71 | 1.4 | 1.11 | 1.765 |
|  | Pancreatic neoplasm | 5 | 5.64 | 2.34 | 13.59 |
|  | Papillary serous endometrial carcinoma | 5 | 456 | 153 | 1360 |
|  | Papillary thyroid cancer | 4 | 3.28 | 1.23 | 8.748 |
|  | Rectal adenocarcinoma | 4 | 8.5 | 3.17 | 22.76 |
|  | Rectal cancer stage IV | 3 | 66.5 | 20.5 | 215.6 |
|  | Renal neoplasm | 5 | 2.75 | 1.14 | 6.617 |
|  | Uterine leiomyoma | 9 | 2 | 1.04 | 3.846 |
| Nervous system disorders | Anosmia | 35 | 4.93 | 3.54 | 6.881 |
|  | Bell's palsy | 8 | 9.66 | 4.81 | 19.41 |
|  | Brain stem haemorrhage | 3 | 4.56 | 1.47 | 14.2 |
|  | Cerebellar infarction | 3 | 3.19 | 1.03 | 9.902 |
|  | Cerebral thrombosis | 7 | 3.91 | 1.86 | 8.208 |
|  | Cerebral ventricle dilatation | 5 | 7.37 | 3.06 | 17.79 |
|  | Cerebrovascular accident | 118 | 1.29 | 1.08 | 1.551 |
|  | Dementia Alzheimer's type | 12 | 1.93 | 1.1 | 3.407 |
|  | Diabetic coma | 5 | 5.21 | 2.16 | 12.55 |
|  | Facial paralysis | 17 | 1.78 | 1.1 | 2.863 |
|  | Head discomfort | 36 | 2.36 | 1.7 | 3.281 |
|  | Headache | 839 | 1.83 | 1.71 | 1.967 |
|  | Hypersomnia | 32 | 1.53 | 1.08 | 2.162 |
|  | Hypotonia | 13 | 2.09 | 1.21 | 3.607 |
|  | Intracranial pressure increased | 8 | 2.25 | 1.13 | 4.512 |
|  | Lacunar infarction | 4 | 3.77 | 1.41 | 10.06 |
|  | Loss of consciousness | 107 | 1.33 | 1.1 | 1.608 |
|  | Mononeuropathy multiplex | 9 | 35.2 | 18 | 68.52 |
|  | Myasthenia gravis | 11 | 2.47 | 1.37 | 4.466 |
|  | Nerve compression | 21 | 2.76 | 1.8 | 4.241 |
|  | Pachymeningitis | 7 | 35.9 | 16.8 | 76.5 |
|  | Paraplegia | 5 | 2.42 | 1.01 | 5.82 |
|  | Peripheral nerve lesion | 3 | 10.2 | 3.28 | 32.01 |
|  | Post polio syndrome | 3 | 79.4 | 24.3 | 259.6 |
|  | Sciatica | 23 | 1.85 | 1.23 | 2.786 |
|  | Speech disorder | 55 | 1.51 | 1.16 | 1.969 |
|  | Syncope | 118 | 1.75 | 1.46 | 2.094 |
|  | Thalamus haemorrhage | 5 | 6.42 | 2.66 | 15.47 |
|  | Transient aphasia | 3 | 26.7 | 8.47 | 84.44 |
|  | Transient ischaemic attack | 35 | 1.93 | 1.39 | 2.695 |
|  | Trigeminal nerve disorder | 3 | 11.8 | 3.78 | 36.97 |
| Product issues | Product availability issue | 140 | 10.1 | 8.58 | 11.99 |
|  | Product complaint | 274 | 14.1 | 12.5 | 15.88 |
|  | Product supply issue | 11 | 1.86 | 1.03 | 3.362 |
| Psychiatric disorders | Discouragement | 11 | 4.49 | 2.48 | 8.121 |
|  | Excessive masturbation | 3 | 28.3 | 8.94 | 89.39 |
|  | Fear of death | 4 | 3.26 | 1.22 | 8.705 |
|  | Frustration tolerance decreased | 16 | 1.74 | 1.07 | 2.844 |
|  | Middle insomnia | 35 | 2.68 | 1.93 | 3.743 |
|  | Near death experience | 8 | 3 | 1.5 | 6.005 |
|  | Nervousness | 66 | 1.85 | 1.45 | 2.354 |
|  | Panic disorder | 8 | 2.94 | 1.47 | 5.894 |
|  | Poor quality sleep | 29 | 1.71 | 1.19 | 2.462 |
|  | Sleep disorder due to a general medical condition | 591 | 59.7 | 54.8 | 64.97 |
|  | Stress | 82 | 1.38 | 1.11 | 1.717 |
|  | Thought blocking | 6 | 18.4 | 8.17 | 41.24 |
| Renal and urinary disorders | Bladder cyst | 4 | 31.2 | 11.5 | 84.8 |
|  | Bladder mass | 3 | 8.23 | 2.64 | 25.66 |
|  | Bladder pain | 12 | 5.13 | 2.91 | 9.051 |
|  | Dysuria | 38 | 1.45 | 1.05 | 1.994 |
|  | Nephrolithiasis | 68 | 1.86 | 1.47 | 2.363 |
|  | Renal artery stenosis | 3 | 3.2 | 1.03 | 9.941 |
|  | Renal colic | 9 | 5.48 | 2.85 | 10.57 |
|  | Renal pain | 17 | 2.1 | 1.3 | 3.378 |
|  | Urinary tract pain | 5 | 12.6 | 5.2 | 30.43 |
| Reproductive system and breast disorders | Bladder prolapse | 5 | 4.42 | 1.83 | 10.64 |
|  | Breast calcifications | 3 | 6.27 | 2.01 | 19.54 |
|  | Breast disorder | 4 | 2.82 | 1.05 | 7.515 |
|  | Ovarian disorder | 3 | 5.05 | 1.62 | 15.72 |
|  | Prostatitis | 8 | 3.72 | 1.86 | 7.455 |
| Respiratory, thoracic and mediastinal disorders | Allergic cough | 3 | 3.84 | 1.24 | 11.95 |
|  | Allergic respiratory disease | 7 | 68.3 | 31.6 | 147.8 |
|  | Allergic sinusitis | 6 | 8.65 | 3.87 | 19.33 |
|  | Aphonia | 43 | 3.99 | 2.95 | 5.385 |
|  | Apnoea | 27 | 5.97 | 4.09 | 8.725 |
|  | Apparent life threatening event | 3 | 14 | 4.46 | 43.76 |
|  | Asphyxia | 12 | 1.92 | 1.09 | 3.386 |
|  | Aspiration | 17 | 2.7 | 1.68 | 4.353 |
|  | Aspirin-exacerbated respiratory disease | 3 | 3.54 | 1.14 | 11.02 |
|  | Asthma | 2804 | 41.4 | 39.7 | 43.16 |
|  | Asthma exercise induced | 7 | 23 | 10.8 | 48.68 |
|  | Asthma-chronic obstructive pulmonary disease overlap syndrome | 9 | 17.3 | 8.93 | 33.46 |
|  | Asthmatic crisis | 714 | 239 | 219 | 259.9 |
|  | Bronchial disorder | 9 | 6.37 | 3.31 | 12.29 |
|  | Bronchial hyperreactivity | 10 | 10.1 | 5.43 | 18.9 |
|  | Bronchial obstruction | 41 | 36.2 | 26.4 | 49.47 |
|  | Bronchial secretion retention | 15 | 12.7 | 7.63 | 21.16 |
|  | Bronchial wall thickening | 15 | 17.9 | 10.8 | 29.94 |
|  | Bronchiectasis | 59 | 10.3 | 7.98 | 13.36 |
|  | Bronchitis chronic | 8 | 3.98 | 1.99 | 7.981 |
|  | Bronchomalacia | 4 | 22.8 | 8.43 | 61.53 |
|  | Bronchopulmonary dysplasia | 3 | 7.01 | 2.25 | 21.84 |
|  | Bronchospasm | 101 | 11.3 | 9.3 | 13.8 |
|  | Catarrh | 17 | 20.7 | 12.8 | 33.46 |
|  | Choking | 27 | 1.9 | 1.3 | 2.777 |
|  | Choking sensation | 28 | 7.32 | 5.04 | 10.62 |
|  | Chronic eosinophilic rhinosinusitis | 5 | 171 | 65.2 | 447.8 |
|  | Chronic obstructive pulmonary disease | 212 | 5.84 | 5.1 | 6.692 |
|  | Cough | 1348 | 6.56 | 6.2 | 6.941 |
|  | Diaphragmatic disorder | 3 | 5.57 | 1.79 | 17.32 |
|  | Dysphonia | 150 | 3.4 | 2.89 | 3.993 |
|  | Dyspnoea | 2879 | 8.74 | 8.38 | 9.104 |
|  | Dyspnoea exertional | 246 | 7.48 | 6.59 | 8.491 |
|  | Emphysema | 37 | 5.5 | 3.98 | 7.606 |
|  | Eosinophilic pneumonia | 6 | 3.14 | 1.41 | 7.01 |
|  | Eosinophilic pneumonia chronic | 3 | 10.4 | 3.32 | 32.42 |
|  | Haemoptysis | 45 | 2.11 | 1.58 | 2.833 |
|  | Hypopnoea | 8 | 3.18 | 1.59 | 6.363 |
|  | Hypoventilation | 20 | 7.9 | 5.09 | 12.28 |
|  | Hypoxia | 45 | 1.88 | 1.4 | 2.522 |
|  | Increased bronchial secretion | 48 | 25 | 18.8 | 33.36 |
|  | Increased upper airway secretion | 21 | 10.1 | 6.58 | 15.56 |
|  | Increased viscosity of bronchial secretion | 17 | 34.9 | 21.5 | 56.69 |
|  | Increased viscosity of upper respiratory secretion | 8 | 11 | 5.45 | 22.01 |
|  | Irregular breathing | 5 | 8.4 | 3.48 | 20.28 |
|  | Laryngeal oedema | 9 | 2.58 | 1.34 | 4.966 |
|  | Lower respiratory tract congestion | 15 | 5.89 | 3.54 | 9.784 |
|  | Lung diffusion disorder | 3 | 18.9 | 6.02 | 59.44 |
|  | Lung disorder | 193 | 5.29 | 4.59 | 6.098 |
|  | Lung hyperinflation | 14 | 16.4 | 9.67 | 27.86 |
|  | Lung infiltration | 24 | 5.8 | 3.88 | 8.666 |
|  | Lung opacity | 17 | 9.28 | 5.75 | 14.97 |
|  | Mouth breathing | 3 | 11.4 | 3.64 | 35.59 |
|  | Nasal congestion | 247 | 5.3 | 4.68 | 6.019 |
|  | Nasal crusting | 7 | 10.4 | 4.94 | 21.96 |
|  | Nasal discharge discolouration | 5 | 2.97 | 1.23 | 7.15 |
|  | Nasal disorder | 9 | 3.84 | 2 | 7.402 |
|  | Nasal dryness | 9 | 1.93 | 1 | 3.715 |
|  | Nasal inflammation | 6 | 6.69 | 2.99 | 14.93 |
|  | Nasal mucosal discolouration | 3 | 25.4 | 8.04 | 80.02 |
|  | Nasal obstruction | 9 | 6.69 | 3.47 | 12.89 |
|  | Nasal oedema | 9 | 8.5 | 4.41 | 16.4 |
|  | Nasal polyps | 62 | 21.3 | 16.5 | 27.37 |
|  | Nasal septum deviation | 7 | 5.12 | 2.43 | 10.76 |
|  | Nocturnal dyspnoea | 10 | 9.87 | 5.29 | 18.42 |
|  | Obstructive airways disorder | 178 | 14.6 | 12.6 | 16.91 |
|  | Obstructive sleep apnoea syndrome | 6 | 15.2 | 6.77 | 34.06 |
|  | Oropharyngeal discomfort | 43 | 6.2 | 4.59 | 8.37 |
|  | Oropharyngeal pain | 182 | 2.38 | 2.06 | 2.756 |
|  | Orthopnoea | 6 | 2.65 | 1.19 | 5.912 |
|  | Painful respiration | 14 | 7.65 | 4.52 | 12.95 |
|  | Pharyngeal erythema | 7 | 4.67 | 2.22 | 9.813 |
|  | Pharyngeal swelling | 20 | 2.61 | 1.68 | 4.048 |
|  | Pharyngeal ulceration | 4 | 3.81 | 1.43 | 10.17 |
|  | Pleural thickening | 4 | 4.77 | 1.78 | 12.74 |
|  | Pleurisy | 10 | 2.45 | 1.32 | 4.562 |
|  | Pleuritic pain | 7 | 4.41 | 2.1 | 9.269 |
|  | Pneumonitis | 48 | 2.33 | 1.76 | 3.101 |
|  | Pneumothorax | 47 | 4.02 | 3.02 | 5.354 |
|  | Productive cough | 467 | 10.9 | 9.93 | 11.96 |
|  | Prolonged expiration | 10 | 36.1 | 19.2 | 68.1 |
|  | Pulmonary congestion | 96 | 10.4 | 8.49 | 12.71 |
|  | Pulmonary embolism | 88 | 1.64 | 1.33 | 2.028 |
|  | Pulmonary fibrosis | 25 | 2.01 | 1.36 | 2.974 |
|  | Pulmonary mass | 55 | 4.25 | 3.26 | 5.539 |
|  | Pulmonary oedema | 72 | 2.41 | 1.91 | 3.042 |
|  | Pulmonary pain | 20 | 6.64 | 4.28 | 10.32 |
|  | Pulmonary thrombosis | 27 | 3.17 | 2.17 | 4.628 |
|  | Rales | 34 | 6.76 | 4.82 | 9.48 |
|  | Respiration abnormal | 56 | 9.84 | 7.56 | 12.81 |
|  | Respiratory depth decreased | 3 | 76.9 | 23.5 | 251.1 |
|  | Respiratory disorder | 128 | 6.01 | 5.05 | 7.162 |
|  | Respiratory distress | 44 | 2.54 | 1.89 | 3.416 |
|  | Respiratory failure | 63 | 1.35 | 1.06 | 1.732 |
|  | Respiratory fatigue | 3 | 13.4 | 4.29 | 42.07 |
|  | Respiratory gas exchange disorder | 6 | 16.1 | 7.17 | 36.08 |
|  | Respiratory symptom | 49 | 19.9 | 15 | 26.45 |
|  | Respiratory tract congestion | 50 | 4.26 | 3.23 | 5.632 |
|  | Respiratory tract inflammation | 4 | 8.84 | 3.3 | 23.68 |
|  | Respiratory tract irritation | 5 | 7.23 | 3 | 17.44 |
|  | Restrictive pulmonary disease | 10 | 10 | 5.38 | 18.74 |
|  | Reversible airways obstruction | 13 | 18 | 10.4 | 31.28 |
|  | Rhinitis allergic | 20 | 6.02 | 3.87 | 9.342 |
|  | Rhinorrhoea | 163 | 2.94 | 2.52 | 3.433 |
|  | Rhonchi | 6 | 5.15 | 2.31 | 11.49 |
|  | Sinus congestion | 35 | 3.45 | 2.48 | 4.814 |
|  | Sinus disorder | 65 | 4.03 | 3.15 | 5.142 |
|  | Sinus pain | 6 | 2.27 | 1.02 | 5.048 |
|  | Sinus polyp | 4 | 8.7 | 3.25 | 23.31 |
|  | Sleep apnoea syndrome | 43 | 2.45 | 1.82 | 3.309 |
|  | Small airways disease | 7 | 87 | 39.9 | 189.6 |
|  | Sneezing | 78 | 4.75 | 3.8 | 5.944 |
|  | Snoring | 14 | 4.63 | 2.74 | 7.828 |
|  | Sputum discoloured | 214 | 20.2 | 17.6 | 23.14 |
|  | Sputum increased | 22 | 11.3 | 7.39 | 17.16 |
|  | Sputum retention | 10 | 12.8 | 6.83 | 23.83 |
|  | Status asthmaticus | 7 | 25.5 | 12 | 54.15 |
|  | Stridor | 12 | 7 | 3.96 | 12.35 |
|  | Suffocation feeling | 14 | 8.25 | 4.87 | 13.96 |
|  | Tachypnoea | 27 | 3.06 | 2.1 | 4.466 |
|  | Throat clearing | 28 | 7.88 | 5.43 | 11.43 |
|  | Throat irritation | 65 | 2.1 | 1.65 | 2.684 |
|  | Throat tightness | 38 | 2.25 | 1.64 | 3.098 |
|  | Tracheal disorder | 3 | 7.86 | 2.52 | 24.5 |
|  | Tracheal stenosis | 5 | 15.4 | 6.34 | 37.2 |
|  | Upper respiratory tract congestion | 8 | 3.89 | 1.94 | 7.801 |
|  | Upper respiratory tract inflammation | 4 | 2.93 | 1.1 | 7.817 |
|  | Upper-airway cough syndrome | 37 | 5.22 | 3.78 | 7.216 |
|  | Use of accessory respiratory muscles | 8 | 31.5 | 15.6 | 63.94 |
|  | Vocal cord disorder | 6 | 4.62 | 2.07 | 10.31 |
|  | Vocal cord dysfunction | 6 | 8.93 | 3.99 | 19.97 |
|  | Wheezing | 1445 | 34 | 32.2 | 35.92 |
| Skin and subcutaneous tissue disorders | Cellulite | 3 | 15.5 | 4.94 | 48.49 |
|  | Dermal cyst | 6 | 3.14 | 1.41 | 6.992 |
|  | Eczema | 79 | 3.13 | 2.51 | 3.903 |
|  | Hand dermatitis | 3 | 8.54 | 2.74 | 26.64 |
|  | Nail bed disorder | 3 | 8.15 | 2.61 | 25.4 |
|  | Pemphigoid | 16 | 2.8 | 1.72 | 4.581 |
|  | Pruritus | 318 | 1.29 | 1.15 | 1.437 |
|  | Pruritus generalised | 34 | 2.44 | 1.74 | 3.414 |
|  | Purpura | 12 | 2.34 | 1.33 | 4.133 |
|  | Pustular psoriasis | 5 | 2.46 | 1.02 | 5.918 |
|  | Rash erythematous | 39 | 1.43 | 1.05 | 1.965 |
|  | Rash pruritic | 53 | 1.52 | 1.16 | 1.993 |
|  | Skin atrophy | 19 | 4.21 | 2.68 | 6.613 |
|  | Skin discharge | 4 | 16.5 | 6.12 | 44.36 |
|  | Skin disorder | 41 | 1.61 | 1.18 | 2.185 |
|  | Skin fragility | 10 | 3.9 | 2.1 | 7.268 |
|  | Skin lesion | 30 | 1.44 | 1.01 | 2.06 |
|  | Urticaria | 167 | 1.52 | 1.31 | 1.774 |
| Social circumstances | Bedridden | 36 | 3.86 | 2.78 | 5.36 |
|  | Cardiac assistance device user | 3 | 13 | 4.16 | 40.72 |
|  | Dependence on oxygen therapy | 9 | 21.6 | 11.2 | 41.98 |
|  | Loss of personal independence in daily activities | 928 | 19.2 | 17.9 | 20.52 |
|  | Social problem | 164 | 61.1 | 52.1 | 71.7 |
|  | Walking aid user | 14 | 3.02 | 1.79 | 5.113 |
|  | Walking disability | 5 | 2.9 | 1.2 | 6.968 |
|  | Wheelchair user | 8 | 2.26 | 1.13 | 4.518 |
| Surgical and medical procedures | Abdominal cavity drainage | 3 | 6.85 | 2.2 | 21.35 |
|  | Abscess drainage | 3 | 6.07 | 1.95 | 18.91 |
|  | Appendicectomy | 7 | 3.38 | 1.61 | 7.1 |
|  | Arterial angioplasty | 3 | 117 | 34.9 | 392.8 |
|  | Biliary tract operation | 3 | 102 | 30.9 | 340.4 |
|  | Bladder catheterisation | 3 | 4.51 | 1.45 | 14.01 |
|  | Bladder operation | 3 | 3.68 | 1.18 | 11.43 |
|  | Breast operation | 4 | 10.1 | 3.78 | 27.14 |
|  | Bronchoplasty | 5 | 205 | 76.9 | 546.3 |
|  | Cancer surgery | 3 | 3.52 | 1.13 | 10.95 |
|  | Cardiac ablation | 4 | 2.91 | 1.09 | 7.762 |
|  | Cardiac operation | 23 | 4.31 | 2.86 | 6.488 |
|  | Cardiac pacemaker insertion | 24 | 6.56 | 4.39 | 9.812 |
|  | Cataract operation | 27 | 7.38 | 5.05 | 10.78 |
|  | Central venous catheterisation | 7 | 2.81 | 1.34 | 5.908 |
|  | Chemotherapy | 6 | 2.35 | 1.05 | 5.234 |
|  | Corneal transplant | 5 | 11.1 | 4.57 | 26.71 |
|  | Coronary arterial stent insertion | 8 | 4.44 | 2.22 | 8.901 |
|  | Coronary artery bypass | 5 | 2.52 | 1.05 | 6.063 |
|  | Drug implantation | 3 | 492 | 118 | 2059 |
|  | Ear operation | 3 | 14.1 | 4.51 | 44.27 |
|  | Emergency care | 15 | 6.6 | 3.97 | 10.97 |
|  | Endotracheal intubation | 16 | 9.27 | 5.66 | 15.18 |
|  | Enterostomy | 4 | 14.5 | 5.4 | 39.01 |
|  | Eye operation | 10 | 3.4 | 1.83 | 6.324 |
|  | Facetectomy | 3 | 410 | 103 | 1639 |
|  | Finger amputation | 3 | 5.63 | 1.81 | 17.52 |
|  | Gastrointestinal surgery | 3 | 5.2 | 1.67 | 16.18 |
|  | Glaucoma surgery | 4 | 23.1 | 8.55 | 62.4 |
|  | High frequency ablation | 4 | 12 | 4.46 | 32.13 |
|  | Hip arthroplasty | 21 | 1.88 | 1.23 | 2.893 |
|  | Hip surgery | 19 | 5.21 | 3.32 | 8.177 |
|  | Hospitalisation | 677 | 5.94 | 5.49 | 6.415 |
|  | Ileostomy | 6 | 3.67 | 1.65 | 8.189 |
|  | Implantable defibrillator insertion | 5 | 7.61 | 3.15 | 18.35 |
|  | Intestinal operation | 14 | 14.9 | 8.78 | 25.28 |
|  | Intraocular lens implant | 3 | 8.72 | 2.8 | 27.21 |
|  | Joint fluid drainage | 4 | 17 | 6.31 | 45.75 |
|  | Joint stabilisation | 3 | 98.4 | 29.7 | 325.9 |
|  | Knee arthroplasty | 24 | 1.62 | 1.09 | 2.425 |
|  | Knee operation | 25 | 3.89 | 2.63 | 5.768 |
|  | Limb operation | 10 | 2.86 | 1.54 | 5.331 |
|  | Lung lobectomy | 3 | 12.8 | 4.1 | 40.08 |
|  | Lung operation | 3 | 5.39 | 1.73 | 16.79 |
|  | Medical induction of coma | 3 | 8.94 | 2.87 | 27.91 |
|  | Myomectomy | 5 | 47.7 | 19.3 | 117.5 |
|  | Nasal polypectomy | 7 | 76.6 | 35.3 | 166.1 |
|  | Nasal septal operation | 5 | 21.7 | 8.92 | 52.74 |
|  | Neck surgery | 9 | 4.92 | 2.55 | 9.473 |
|  | Nephrectomy | 6 | 5.27 | 2.36 | 11.76 |
|  | Oxygen therapy | 10 | 6.49 | 3.48 | 12.09 |
|  | Palliative care | 12 | 12 | 6.81 | 21.29 |
|  | Paracentesis | 3 | 5.18 | 1.66 | 16.12 |
|  | Patient isolation | 25 | 95.9 | 63.4 | 145.2 |
|  | Polypectomy | 9 | 17.2 | 8.87 | 33.23 |
|  | Prostatectomy | 3 | 8.94 | 2.87 | 27.91 |
|  | Quarantine | 39 | 140 | 99.7 | 196.7 |
|  | Renal artery angioplasty | 3 | 145 | 42.4 | 493.8 |
|  | Renal artery stent placement | 3 | 30 | 9.48 | 94.95 |
|  | Renal stone removal | 6 | 14.4 | 6.44 | 32.35 |
|  | Renal surgery | 4 | 9.34 | 3.49 | 25.04 |
|  | Retinopexy | 3 | 63.1 | 19.5 | 204.1 |
|  | Self-medication | 8 | 4.54 | 2.27 | 9.099 |
|  | Shoulder operation | 7 | 2.46 | 1.17 | 5.157 |
|  | Sinus operation | 45 | 26.7 | 19.8 | 35.95 |
|  | Spinal anaesthesia | 4 | 82 | 29.3 | 229.2 |
|  | Spinal decompression | 3 | 11.3 | 3.61 | 35.26 |
|  | Stent placement | 17 | 3.47 | 2.16 | 5.593 |
|  | Stoma closure | 4 | 20.4 | 7.55 | 54.95 |
|  | Surgery | 65 | 1.75 | 1.37 | 2.231 |
|  | Suture insertion | 4 | 10.3 | 3.85 | 27.66 |
|  | Therapy cessation | 72 | 1.62 | 1.28 | 2.042 |
|  | Therapy interrupted | 149 | 4.42 | 3.76 | 5.191 |
|  | Toe operation | 3 | 5.65 | 1.82 | 17.6 |
|  | Tonsillectomy | 6 | 6.53 | 2.93 | 14.59 |
|  | Tooth extraction | 21 | 2.64 | 1.72 | 4.049 |
|  | Tracheostomy | 3 | 3.22 | 1.04 | 10.02 |
|  | Treatment delayed | 3 | 4.92 | 1.58 | 15.31 |
|  | Uterine dilation and curettage | 4 | 9.09 | 3.39 | 24.34 |
|  | Vascular graft | 5 | 4.15 | 1.72 | 9.984 |
| Vascular disorders | Aortic disorder | 3 | 6.34 | 2.04 | 19.75 |
|  | Aortic stenosis | 8 | 3.98 | 1.99 | 7.971 |
|  | Arterial occlusive disease | 11 | 2.31 | 1.28 | 4.17 |
|  | Arteriosclerosis | 10 | 2.04 | 1.1 | 3.794 |
|  | Arteriovenous fistula | 3 | 8.06 | 2.59 | 25.15 |
|  | Hyperaemia | 8 | 6.45 | 3.22 | 12.94 |
|  | Hypertension | 178 | 1.18 | 1.02 | 1.369 |
|  | Infarction | 10 | 2.08 | 1.12 | 3.865 |
|  | Jugular vein distension | 3 | 7.69 | 2.47 | 23.96 |
|  | Thrombosis | 78 | 1.4 | 1.12 | 1.745 |
|  | Varicose vein | 13 | 3.39 | 1.96 | 5.839 |
|  | Vascular pain | 6 | 7.24 | 3.24 | 16.17 |
|  | Vasculitis | 31 | 4.41 | 3.1 | 6.286 |
